# Treatment of Tendinous Rheumatism by the External Employment of Sulphur

**Published:** 1864-05

**Authors:** 


### TREATMENT OF TENDINOUS RHEUMATISM BY THE EXTERNAL EMPLOYMENT OF SULPHUR.

Tendinous rheumatism, according to Dr. Renard, differs from acute rheumatism by the absence of the general symptoms, and from the chronic by the presence of local inflammatory symptoms. Dr. Renard suffered from this complaint himself after an attack of acute rheumatism, for which he was copiously bled. The parts affected were the tendons of the hamstring muscles, and no improvement resulted after a long course of diaphoretics, camphor, terebinthinate, and other liniments, and the administration of the solanaceæ. At last Dr. Renard saw a passage in an English medical journal, stating that persons suffering from rheumatism in the legs had only to dust the inside of their stockings with sulphur. He immediately employed this simple remedy, the sulphur being the commercial flowers of brimstone, which contain some sulphurous acid. The curative effect was very well marked, for Dr. Renard walked in the evening, then renewed the sulphur in the stockings before sleeping in them, found himself very much relieved the next morning, and nearly quite cured on the morning after. A few days later he left off the brimstone, and the pain re-appeared in the soles of the feet, but yielded very soon to the re-application of sulphur. Since the year 1857, when he was first attacked, the same experiment was repeated every winter when he was suffering from chronic tenodynia, either in the hams, the heels, or the elbows. He left under the influence of the contact of the flowers of brimstone, the skin becoming hotter, slightly excited, and more disposed to sweating; and, as soon as this effect was produced, the relief of the pain seemed to be immediately marked. Whatever may be the explanation of the manner in which sulphur exerts its curative agency, Dr. Renard affirms that it has a beneficial effect upon the rheumatic pains of the tendons, and that this action is the more rapid and certain in proportion as the tendons are more superficial and the sulphur is kept more closely over the painful parts.—*B. & F. Med. Chir. Rev.*, Jan. 1864, from *L'Union Medicale*, April 21, 1863.—*Chicago Examiner.*

